# Mechanism for the Magnetorheological Effect of Nanocomposite Hydrogels with Magnetite Microparticles

**DOI:** 10.3390/gels9030218

**Published:** 2023-03-14

**Authors:** Lukas Selzer, Stefan Odenbach

**Affiliations:** Institute of Mechatronic Engineering, Technische Universität Dresden, George-Bähr-Str. 3, 01062 Dresden, Germany

**Keywords:** nanocomposite hydrogel, NIPAAm, laponite, microparticles, magnetite, magnetorheological effect, tomography

## Abstract

In a previous study, we presented an empirical law for the magnetorheological effect of nanocomposite hydrogels with magnetite microparticles derived from rheological data. In order to understand the underlying processes, we employ computed tomography for structure analysis. This allows the evaluation of the translational and rotational movement of the magnetic particles. Gels with 10% and 3.0% magnetic particle mass content are investigated at three degrees of swelling and at different magnetic flux densities in steady states by means of computed tomography. Since a temperature-controlled sample-chamber is difficult to implement in a tomographic setup, salt is used to deswell the gels instead. Based on the findings of the particle movement, we propose a mechanism using an energy-based approach. This leads to a theoretical law that shows the same scaling behavior as the previously found empirical law.

## 1. Introduction

While empirical laws offer some insight into how a material will react, they do not provide an underlying mechanism on how phenomena occur. Computed tomography is a method that allows investigating the structure of a material in a non-destructive way, which has led to its main use in medicine [1]. It was shown for magnetoactive elastomers that the movement and structure formation of the magnetic particles under the influence of a magnetic field are linked to the mechanical properties of the material [2].

In our previous study [3], we demonstrated an empirical law for the magnetorheological effect in nanocomposite hydrogels with magnetite microparticles. Nanocomposite hydrogels can be synthesized by polymerizing *N*-isopropylacrylamide in the presence of inorganic clay as a crosslinking agent, which results in more mechanically robust gels when compared to hydrogels synthesized with organic crosslinking agents [4]. Additionally, the sedimentation of magnetic microparticles during the synthesis of magnetic nanocomposite hydrogels can be prevented by the impact of the clay particles on the rheological properties of the reaction mixture [3].

In this study, we now investigate these magnetic gels by means of computed tomography. By comparing the angle distributions of particles in steady states of the material under the influence of magnetic fields with varying flux densities, we investigate the impact of the magnetic field on the mean particle angle, as well as the reversibility of this rotational movement. By determining the distance to the nearest neighbor of the particles, statistical information about translational movement can be gained. When only the nearest neighbor within a double cone along an axis is considered instead, information about anisotropic movement can be obtained. Using different degrees of swelling shows how the particle concentration and the mechanical baseline properties of the material impact the movement of the particles.

While we used temperature as the stimulus in our previous study, we use salt for deswelling in this study, as we could reach similar degrees of swelling and mechanical properties by using either stimulus, and as this simplified the requirements for the tomographic sample chamber. Based on the findings of the particle movement, we propose a mechanism using an energy-based approach.

## 2. Materials and Methods

### 2.1. Materials

Magnetite particles 48806 (98.7%, *ρ* = 5.17 g cm^−3^) were purchased by Kremer Pigmente, Aichstetten, Germany and sieved before usage. For deionized water, an ultrapure water filter PRO VE 3+ by AFT, Zirndorf, Germany was used. Tetramethylethylenediamine (99%) and sodium persulfate (≥98.0%) were purchased from Sigma-Aldrich, Taufkirchen, Germany and *N*-isopropylacrylamide (stabilized by Mequinol, ≥98.0%) was purchased from Tokyo Chemical Industry, Eschborn, Germany. Laponite RD and Laponite RDS were obtained by BYK, Wesel, Germany. All chemicals were used without further purification.

### 2.2. Sieving of Particles

Magnetite particles were dry sieved using a sieving tower by Haver & Boecker, Oelde, Germany. Sieves with mesh sizes of 25, 50, 100 and 200 μm were stacked, and an amplitude of 3 mm was used for 15 min. The fraction from 50 to 100 μm of 200 g magnetite powder was collected, sieved again using the same setup and used for the synthesis.

### 2.3. Particle Size Distribution

The particle size distribution of the particle fraction from 50 to 100 μm was determined by laser scattering using a HELOS/KR-H2487 by Sympatec, Clausthal-Zellerfeld, Germany with a RODOS dispersion unit. The measurements were conducted by the group for Mechanical Process Engineering at Technische Universität Dresden, Dresden, Germany.

### 2.4. Synthesis of Magnetorheological Nanocomposite Hydrogels

Deionized water (27 mL) was placed into a 50 mL beaker and stirred at 600 min^−1^. Tetramethylethylenediamine (72 μL) and *N*-isopropylacrylamide (3 g) were added. After this dissolved, Laponit RDS (1 g) was added. After this was dissolved, Laponit RD (2 g) was added slowly, and the solution was stirred at 800 min^−1^ until everything was dissolved. The solution was then picked up into two 20 mL syringes and sonicated in an Elmasonic P 60 H by Elma for 1 min at 37 kHz to gather and remove air bubbles. This pregel-solution was stored for 12 h at room temperature before use. Deionized water (3 mL) was placed into a 5 mL Eppendorf tube. Sodium persulfate (94.6 mg) was added and mixed by inversion. This initiator-solution was prepared directly before use.

For the synthesis of a hydrogel, the pregel-solution (5 g) was added to a 10 mL syringe. Magnetite particles were added according to Table 1. Using a vortex mixer, the suspension was homogenized. Afterward, the initatior-solution (0.45 g) was added into the syringe. The suspension was again homogenized using a vortex mixer and then injected into a mold with inner dimensions of 45 mm × 45 mm × 2 mm. After 24 h at room temperature, the polymerization reaction was completed.

### 2.5. Storage of Gels

The gels were stored in 200 mL deionized water as storage solution within waterproof plastic vessels. These were placed into a water-bath WNB14 by Memmert for 14 days at 20 °C. During this time, the storage solution was refreshed daily. Afterward, the samples were cut into equal sized pieces and placed into plastic vessels containing storage solutions with *c*_NaCl_ = 0 mol L^−1^, 0.1 mol L^−1^ and 1 mol L^−1^ and stored for three days at 20 °C. After that, rheological measurements were conducted at 20 °C. The vessels were then stored in the tomographic chamber at 17 °C a day before the tomographic measurements.

### 2.6. Rheological Measurements

Samples of 13 mm diameter were punched out of the gels using a STAS.01 sample punch by Q-tec. The samples were swabbed by a laboratory wipe to remove excess water from the surfaces of the samples. A small film of superglue was applied to the upper and lower measurement geometry. The sample was affixed to the upper geometry and then lowered by the rheometer until there is contact. Afterwards the normal force was set to 0.5 N. The sample was surrounded by 1 mL of storage solution to prevent drifts by evaporation.

Dynamic mechanical analysis was used for all measurements. For all samples, amplitude- and frequency tests were performed to check for correct positioning and fixture. Magnetorheological measurements were performed for 225 s at f=1Hz and γ = 1% for *c*_NaCl_ = 0 mol L^−1^ and 0.1 mol L^−1^ and γ = 0.1% for *c*_NaCl_ = 1 mol L^−1^. The magnetic field was activated at 45 s and deactivated at 135 s. Series of measurements were performed this way for each sample using B= 0 mT to $B=B_{\mathrm{Max}}$ in succession.

### 2.7. Tomographic Measurements

**Figure 1:**
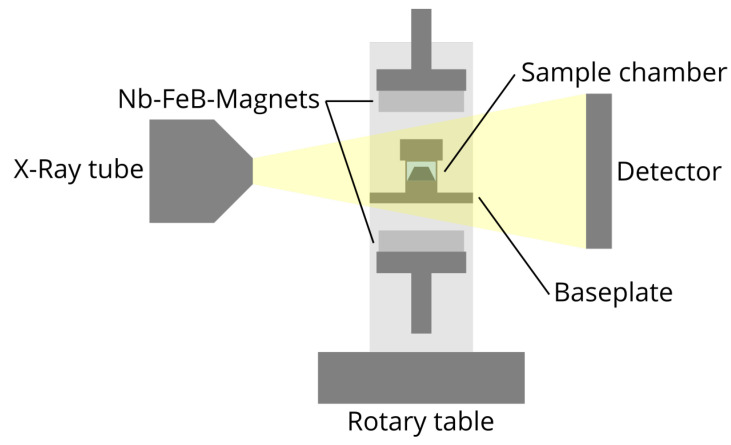
Draft of the tomographic setup. The sample is within a sample chamber with storage solution. Two Nb-FeB-magnets are used to generate a magnetic field at the sample position. The magnetic flux density is adjusted by the distance of the magnets and the sample. The sample holder is placed on a rotary table between the X-ray tube and the detector.

Tomographic measurements were performed using the “TomoTu” lab setup at our chair at TU Dresden. The setup employs a nanofocus X-ray tube XS160NFOF from GE Measurement and Control Solutions with a tungsten target and a flatpanel detector Shad-o-Box 6k GK HS CsI from Teldeyne DALSA with a resolution of 2940 px × 2304 px, a pixel size of 49.5 μm and a digitization of 14 bit. A tube current of I= 170 μA and an acceleration voltage of U= 90 kV were used. Radiograms were taken from 0 ° to 360 ° with 0.25 ° increments. A draft of the setup is shown in Figure 1.

The sample holder was made of two acrylic glass tubes, which were attached to a threaded baseplate. A sample chamber could be attached to the baseplate by a thread connection. Magnets were attached to threaded aluminum sockets, which could adjust the distance of the magnets to the sample.

Spacers were used to ensure symmetrical distance between the sample and both magnets. The resulting magnetic flux densities at the sample position were measured with a 5180 gaussmeter by F.W. Bell. Samples were punched out of the gels manually using a biopsy punch with a 5 mm diameter. The samples were fixed to the bottom of the sample chamber using superglue. The chamber was then immersed in the storage solution of the gel and closed to minimize any air in the chamber.

In total, seven samples were investigated. One sample with $w_{s}$ = 10% stored in *c*_NaCl_ = 0 mol L^−1^ was tomographed successively at B= 0 mT, 45 mT, 90 mT, 200 mT, 380 mT and again at 0 mT. Other samples with $w_{s}$ = 10% and $w_{s}$ = 30% and stored in *c*_NaCl_ = 0 mol L^−1^, *c*_NaCl_ = 0.1 mol L^−1^, and *c*_NaCl_ = 1 mol L^−1^, respectively, were tomographed at B= 0 mT, B= 380 mT and again at B= 0 mT.

Reconstruction was based on the FDK algorithm [5]. The segmentation was based on a python script by Emmanuelle Gouillart, which employs a belief-propagation algorithm [6]. The algorithm was modified to use the Pytorch library, which allowed the usage of GPU for the segmentation and sped up the segmentation significantly [7]. As for the size of the tomograms as well as the limited graphics memory, the segmentation was performed for overlapping subvolumes, which were placed together afterward.

### 2.8. Particle Analysis

Using the sci-kit-image library, the segmentations were labeled and further analyzed [8]. The center positions of all particle regions were used to calculate the distance $d_{\mathrm{NN}}$ from one particle to its nearest neighbor. This was also performed for neighboring particles within a 45° double cone along the *x*-, *y*- and *z*-axis, which yielded $d_{\mathrm{NN},x}$, $d_{\mathrm{NN},y}$ and $d_{\mathrm{NN},z}$, respectively. The equivalent spherical diameter $d_{V}$ of each particle region was calculated.

Using the image moment of each particle region, the lengths of each of the three main axis were obtained and are denoted $d_{I,\max}$, $d_{I,\mathrm{mid}}$ and $d_{I,\min}$, for the longest, the medial and the shortest axis, respectively. By calculating the eigenvectors of each particle region, the orientation angle $\theta_{z}$ of the particle and the *z*-axis was determined. The applied magnetic field was parallel to the *z*-axis. To evaluate changes of the angle distributions, the solid angle has to be considered, which corresponds to the height $h_{\Omega }$ of spherical segments, which can be calculated using:

(1) $$h_{\Omega }=1-\cos\theta_{z}$$

A value of $h_{\Omega }=0$ equals a parallel alignment towards the *z*-axis, while a value of $h_{\Omega }=100$% equals a perpendicular orientation of the particles.

## 3. Results and Discussion

### 3.1. Comparability of Mechanical Properties

Since different stimuli for the deswelling of the gels were used in our studies, first, the comparability of the mechanical properties must be established. Table 2 shows the storage and loss moduli for gels, either deswollen by temperature or by salt. It has to be noted that the batch of this study used particles from 50 to 100 μm instead of 25 to 50 μm, which were used in the previous study. This was necessary for reliable segmentation in the tomographic data.

The different batches showed the same mechanical properties in the swollen state within the precision of the measurements. At the deswollen state, the gels reached approximately the initial volume of the state of synthesis for both cases. $G^{\prime }$ and $G^{\prime \prime }$ were significantly higher in the case of the deswelling by temperature stimulus, which could be attributed either to the stimulus itself or differences between the batches. The values are, however, in the same order of magnitude, and thus results of our tomographic experiments should also hold true for gels of different batches.

### 3.2. Visual Evaluation

**Figure 2:**
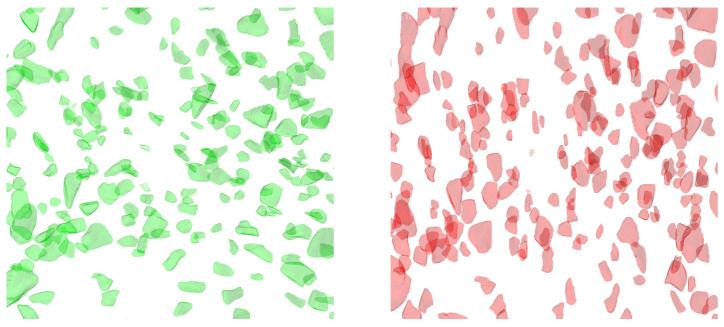
Central cuts of the tomograms of a gel with $w_{s}=10$% and *c*_NaCl_ = 0 mol L^−1^ at B=0 mT (**green**) and B=380 mT (**red**).

Figure 2 shows two central cuts of a gel with $w_{s}=10$% and *c*_NaCl_ = 0 mol L^−1^ at B= 0 mT and B= 380 mT. Qualitatively, the particles are aspherical and oriented perpendicular to the *z*-axis at first without a magnetic field and then parallel to the *z*-axis with a magnetic field. The perpendicular orientation can be attributed to the preparation process of the gels. The reaction mixture fills the mold in the x,y-plane so that an orientation of the long axis of the particles parallel to the flow is preferred, which yields a perpendicular orientation to the *z*-axis.

### 3.3. Particle Size Distribution

**Figure 3:**
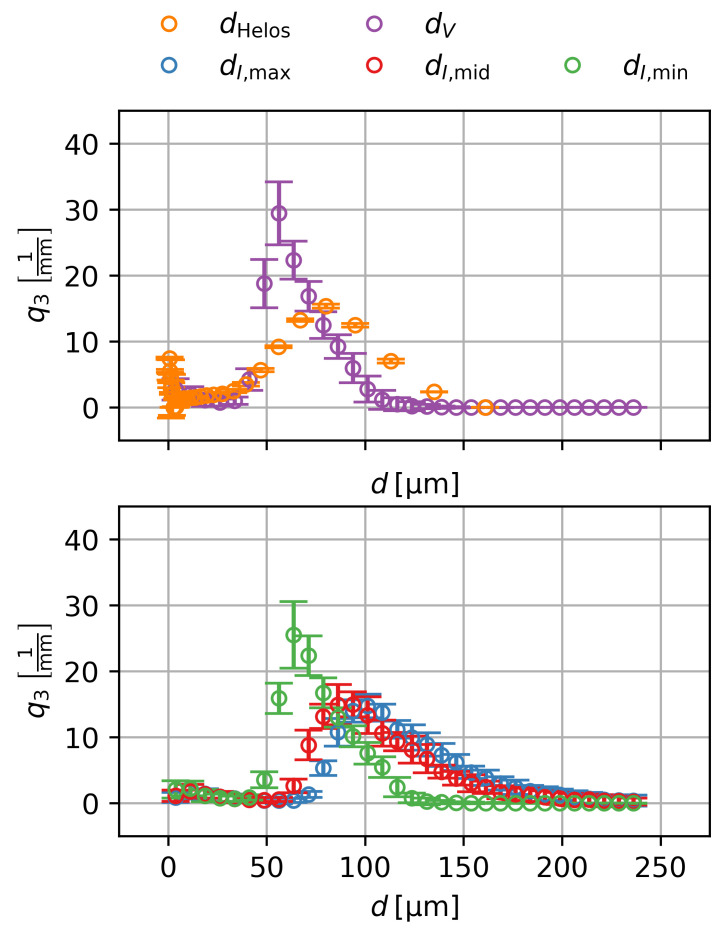
Particle size distributions of the diameters obtained by laser scattering ($d_{\mathrm{Helos}}$), of the equivalent spherical diameters ($d_{V}$), as well as of the diameters obtained through image moments ($d_{I,\max}$, $d_{I,\mathrm{mid}}$ and $d_{I,\min}$).

Figure 3 shows the particle size distributions of the diameters obtained by laser scattering, of the equivalent spherical diameters, as well as of the diameters obtained through image moments. The mean particle diameter obtained by light scattering is $d_{\mathrm{Helos}}=90\pm 72$ μm with a symmetrical distribution, while the particles have a mean equivalent spherical diameter of $d_{V}=70\pm 50$ μm with a positively skewed distribution. The diameters obtained by image moments show that, as seen in Figure 2, the particles have one shorter axis and two larger axes.

Thus, the particles have an oblate geometry. As an average diameter over all three major axis is obtained by laser scattering, and as the distributions of the light scattering diameters and the diameters obtained by image moments are in good agreement, this also shows that the used algorithm yields a good segmentation of the particles. The lower equivalent spherical diameter can be attributed to the aspherical geometry and the surface roughness of the particles. Due to their shape, the sieving is not as effective, leading distributions of the diameters even beyond the nominal cutoff diameter of 100 μm.

### 3.4. Evaluation of the Angle Distribution

**Figure 4:**
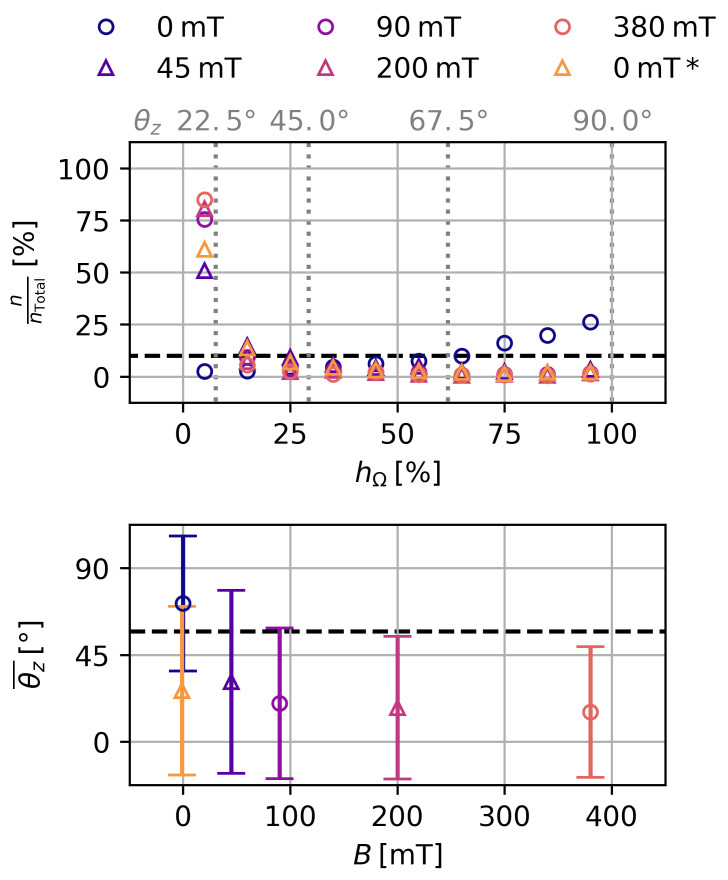
Distribution of the solid angle represented by the relative height of the spherical segment $h_{\Omega }$ and the average *z*-angle $\bar{\theta_{z}}$ over the magnetic flux density. Data are shown for a sample with $w_{s}$ = 10% at *c*_NaCl_ = 0 mol L^−1^. The expected value of 10%, the average *z*-angle $\bar{\theta_{z}}=57{}^{{}^{\circ}}$ for an equal solid angle distribution of the particles as well as some *z*-angles $\theta_{z}$ are shown for orientation.

Figure 4 shows the distribution of the relative height of the spherical segment $h_{\Omega }$, which is proportional to the solid angle, for a sample with $w_{s}=10$% and *c*_NaCl_ = 0 mol L^−1^. In the initial state, the particles show an equal distribution of solid angle of the particles, which is slightly skewed towards $\theta_{z}=90{}^{{}^{\circ}}$ as previously observed in Figure 2. Initially, only 2.5% of all particles were within $h_{\Omega }$ = 0% and $h_{\Omega }$ = 10%. Even with the smallest magnetic flux density of B=45 mT, this value increased sharply to 51% of all particles.

At the maximum magnetic flux density of B=380 mT, 85% of all particles were within this solid angle. Since 61% of all particles were still within this solid angle at B=0 mT afterward, the rotation of the particles was largely irreversible. This is also reflected in the mean particle *z*-angle $\bar{\theta_{z}}$, which decreased inversely with the applied magnetic flux density and did not return to its initial value. The other six samples showed qualitatively the same behavior. The irreversibility can be quantified by the ratio of the initial and the lasting change of the solid angle, with the end state marked as $B=0\;{mT}^{*}$:

(2) $$\frac{\Delta {\bar{h_{\Omega }}}_{0}}{\Delta {\bar{h_{\Omega }}}_{B}}=\frac{\bar{h_{\Omega }}\left(B=0\;mT*\right)-\bar{h_{\Omega }}\left(B=0\;mT\right)}{\bar{h_{\Omega }}\left(B=380\;mT\right)-\bar{h_{\Omega }}\left(B=0\;mT\right)}$$

This, as well as the mean particle *z*-angle ${\bar{\theta_{z}}}_{B}$ at B=380 mT and the storage modulus $G^{\prime }$, is shown in Table 3 for all seven samples. The irreversibility varies from 65% and 92% and shows no clear relationship with other parameters. The average *z*-angle $\bar{\theta_{z}}$ increases with the storage modulus of the sample.

### 3.5. Evaluation of the Particle Distance

**Figure 5:**
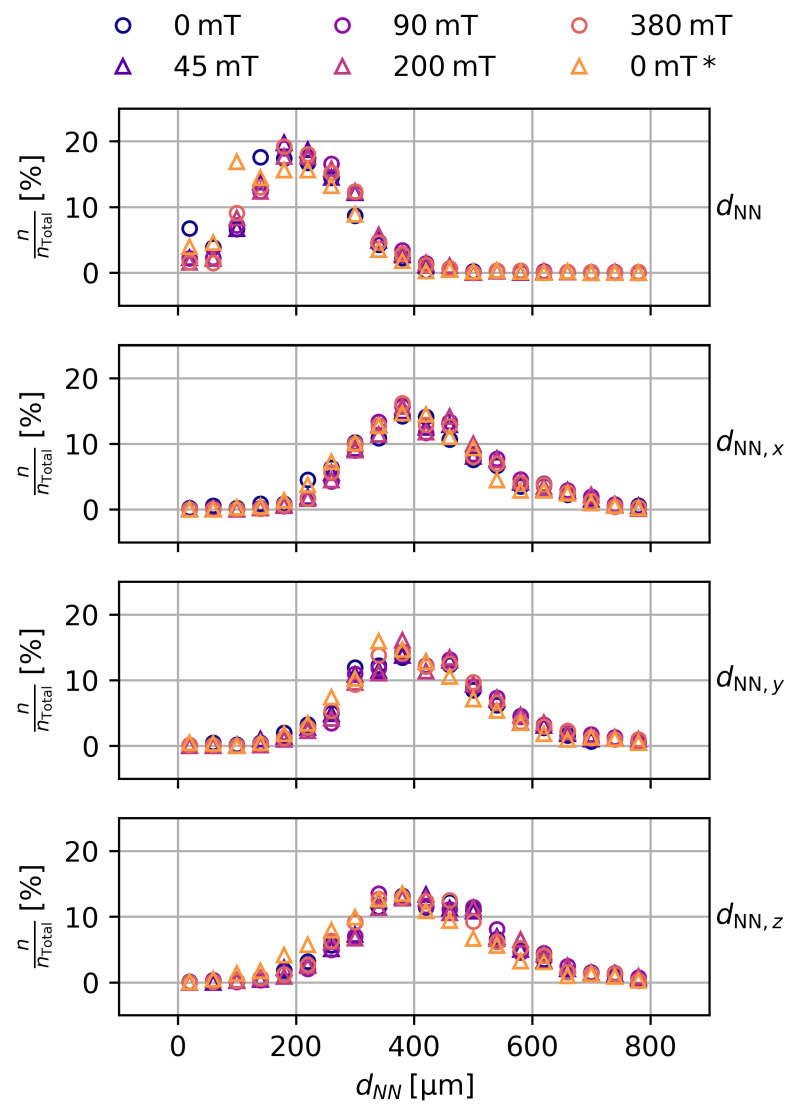
Distributions of the isotropic and anisotropic distances $d_{\mathrm{NN}}$. In the isotropic case, all neighbors of a particle are considered, while only the nearest neighbor within a $45{}^{{}^{\circ}}$ double cone along the *x*-, *y*-, and *z*-axis of a particle are considered in the anisotropic case. Data are shown for a sample with $w_{s}$ = 10% and *c*_NaCl_ = 0 mol L^−1^ with increasing magnetic flux densities.

Figure 5 shows the distribution of the isotropic and anisotropic distances $d_{\mathrm{NN}}$ of particles to their nearest neighbor for a sample with $w_{s}$ = 10% and *c*_NaCl_ = 0 mol L^−1^. In the isotropic case ($d_{\mathrm{NN}}$), the distribution is fairly broad, with a mean value of $d_{\mathrm{NN}}=210\pm 190$ μm. Most importantly, no statistical significant changes of the interparticle distance can be detected at any magnetic flux densities.

Since anisotropic effects, such as chain-formation of the particles due to the magnetic field, are a known phenomenon in magnetorheological elastomers [2], we also checked for the nearest neighbor along the three main axes ($d_{\mathrm{NN},x}$, $d_{\mathrm{NN},y}$ and $d_{\mathrm{NN},z}$). Again, we found no change of the interparticle distance regardless of the applied magnetic flux density. Other samples showed qualitatively the same behavior, with the interparticle distance decreasing with the initial particle content $w_{S}$ and increasing with the degree of swelling of the samples. This was to be expected, as both parameters affect the volume concentration $\phi_{p}$ of the particles inside the samples.

### 3.6. Mechanism

From our rheological study, we obtained the following empirical law for the scaling behavior of the MRE with the Langevin function L(B): [3]

(3) $$\mathrm{MRE}\left(B,\phi_{p},G^{\prime }\right)=\frac{K_{p}\;\cdot \;L\left(B\right)\;\cdot \;\phi_{p}}{\sqrt{G^{\prime }}}$$

**Figure 6:**
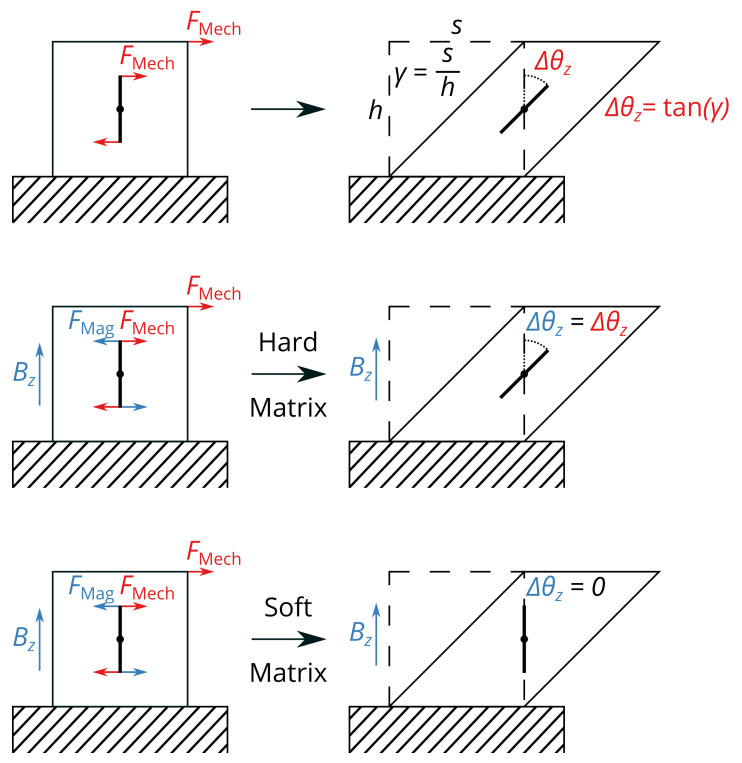
A rod magnet inside an elastic matrix material will rotate by $\Delta \theta_{z}=\tan\left(\gamma \right)$ when the matrix material is sheared by γ. When a magnetic field is applied, two extreme cases for a hard or a soft matrix material can be described, which leads to different rotation angles $\Delta \theta_{z}$.

From our tomographic experiments, we can conclude that the particles are only rotating and do not show translational movement when a magnetic field is applied. While this observation is for steady states, this should also hold true for dynamic states, such as during the rheological measurements. For our mechanism, we assume a very basic model of a rod magnet in an elastic matrix material and a homogeneous magnetic field along the *z*-axis. This is shown in Figure 6. Since the scaling of the MRE in our empirical law is directly proportional to $\phi_{p}$, which implies that the interparticle interaction is negligible for the MRE, all particles can be approximated by a single rod magnet. If the matrix material is sheared by a shear strain γ with no magnetic field active, the rod magnet will follow the movement of the matrix material and, thus, change its initial orientation angle $\theta_{z}$ to the *z*-axis by the rotation angle $\Delta \theta_{z}$.

The rotation angle $\Delta \theta_{z}$ can be calculated by:

(4) $$\Delta \theta_{z}=\tanh\gamma$$

If a magnetic field is applied along the *z*-axis when the matrix material is sheared, two extreme cases can be described. Due to the magnetic field, there is a magnetic torque that keeps the rod aligned with the magnetic field. If the matrix material is hard, the mechanical torque will exceed this magnetic torque and the rod will rotate by the rotation angle $\Delta \theta_{z}$ as before. If the matrix material is very soft, the rod will stay aligned with the magnetic field. Between these extreme cases, the rotation angle will decrease depending on the stiffness of the matrix material. In total, the final angle $\theta_{z}+\Delta \theta_{z}$ of the rod magnet is dependent on the stiffness of the matrix material, the magnetization of the rod magnet, the applied shear and the applied magnetic flux density.

When the matrix is sheared by γ, a shear energy $E_{\mathrm{Mech}}$ is stored in the material. This can be calculated with the storage modulus $G^{\prime }$, the shear strain γ and the volume *V* of the material:

(5) $$E_{\mathrm{Mech}}=-\int Fdx=-\int G^{\prime }\gamma hdAd\gamma =-\frac{1}{2}G^{\prime }\gamma^{2}V$$

On the other hand, if a magnet is rotated out of a magnetic field, the stored energy is dependent on the initial angle $\theta_{z}$ and the final angle $\theta_{z}+\Delta \theta_{z}$. This can be calculated with the magnetic moment *m*, the magnetic flux density *B*, the initial angle $\theta_{z}$ and the rotation angle $\Delta \theta_{z}$:

(6) $$\Delta E_{\mathrm{Mag}}=mB\;\cdot \;\left(\cos\left(\theta_{z}+\Delta \theta_{z}\right)-\cos\left(\theta_{z}\right)\right)$$

In the case of the storage modulus, the magnetorheological effect can be interpreted as the relative increase of the energy stored in the material when it is sheared while a magnetic field is applied. The two extreme cases have to be distinguished. For a very stiff matrix and at low magnetic flux densities, the magnetic rod will completely rotate by Δθ=tanγ, and the additional energy will be the magnetic energy as described by Equation (6). However, for a very soft matrix and at high magnetic flux densities, the rod will stay oriented to the magnetic field during shearing.

This causes additional local stress in the matrix in the vicinity of the rod and, as a result, additional shear energy. This shear energy has to be equal or lower than in Equation (6). Between these extreme cases, the total additional energy stored during shearing, when a magnetic field is present, will be a mixture of magnetic energy stored in the rotation and additional shear energy but can never exceed the energy value of Equation (6), as the magnetic forces are the cause of the additional stored energy. As an upper limit, we can use Equations (5) and (6):

(7) $$E_{\mathrm{Mech}}\;\cdot \;\left(1+\mathrm{MRE}\right)=E_{\mathrm{Mech}}+\Delta E_{\mathrm{Mag}}$$

(8) $$\mathrm{MRE}=\frac{\Delta E_{\mathrm{Mag}}}{E_{\mathrm{Mech}}}$$

For a cube of matrix material with a side length of a=10 mm, a storage modulus of $G^{\prime }=3\;k\mathrm{Pa}$ and a shear of γ = 1%, the stored energy is $E_{\mathrm{Mech}}=0.15$ μJ. On the other hand, for a magnetic rod made out of magnetite with a saturation magnetization of $M_{s}=92$ A m^2^ kg^−1^, [9] a volume corresponding to $\phi_{p}$ =10% of the matrix material and a rotation from $\theta_{z}=0{}^{{}^{\circ}}$ to $\Delta \theta_{z}=\tan\left(\gamma \right)$, the stored magnetic energy is $\Delta E_{\mathrm{Mag}}=0.95$ μJ at B=500 mT. The ratio of these two energies yields a theoretical value of MRE = 643% according to Equation (8). In our experiments and according to our empirical law, gels with the assumed properties show values of MRE = 168%. While the theoretical upper limit of the MRE is larger by a factor of 4, the order of magnitude is correct, which supports this very simple approach as an approximation.

Combining Equations (5), (6) and (8), the scaling behavior of this theoretical MRE can be investigated:

(9) $$\mathrm{MRE}=-\frac{2mB\;\cdot \;\left(\cos\left(\theta_{z}+\Delta \theta_{z}\right)-\cos\left(\theta_{z}\right)\right)}{G^{\prime }\gamma^{2}V}$$

### 3.7. Angular Scaling Behavior

**Figure 7:**
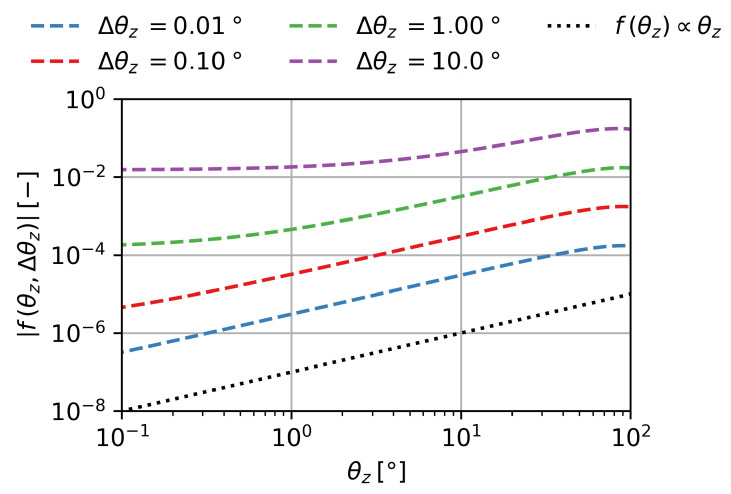
The subfunction $f\left(\theta_{z},\Delta \theta_{z}\right)=\left(\cos\left(\theta_{z}+\Delta \theta_{z}\right)-\cos\left(\theta_{z}\right)\right)$ for various $\Delta \theta_{z}$ over $\theta_{z}$ in double logarithmic scale.

To fully understand the scaling behavior of the MRE with the initial angle $\theta_{z}$ and the rotation angle $\Delta \theta_{z}$, the subfunction $f\left(\theta_{z},\Delta \theta_{z}\right)=\left(\cos\left(\theta_{z}+\Delta \theta_{z}\right)-\cos\left(\theta_{z}\right)\right)$ has to be analyzed. The absolute values of the subfunction are shown in Figure 7 over $\theta_{z}$ for different $\Delta \theta_{z}$ in double logarithmic scale. Absolute values are necessary, as negative values cannot be represented in logarithmic scale. In this depiction, the slope *n* corresponds to the exponent of the relation of $f\left(\theta_{z},\Delta \theta_{z}=\mathrm{const}.\right)\propto {\theta_{z}}^{n}$. For small rotation angles, the term shows a linear scaling behavior with $\theta_{z}$:

(10) $$\lim_{\Delta \theta_{z}\to 0{}^{{}^{\circ}}}f\left(\theta_{z},\Delta \theta_{z}\right)\propto \theta_{z}$$

**Figure 8:**
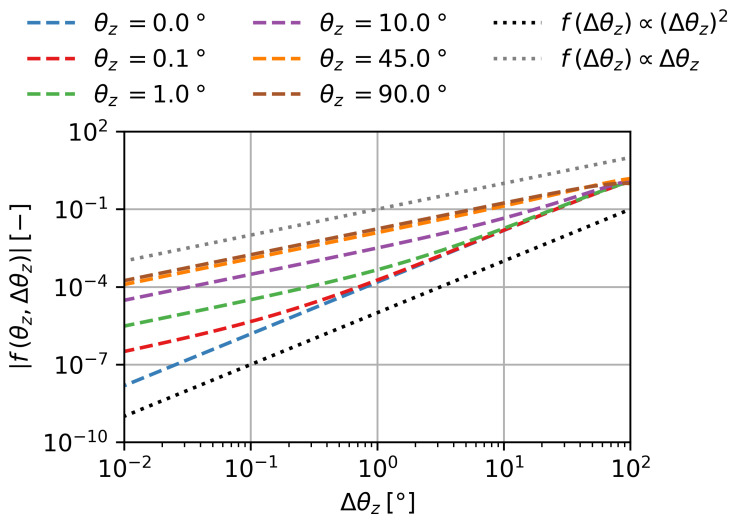
The subfunction $f\left(\theta_{z},\Delta \theta_{z}\right)=\left(\cos\left(\theta_{z}+\Delta \theta_{z}\right)-\cos\left(\theta_{z}\right)\right)$ for various $\theta_{z}$ over $\Delta \theta_{z}$ in double logarithmic scale.

In Figure 8, the absolute values of the subfunction are shown over $\Delta \theta_{z}$ for different $\theta_{z}$ in double logarithmic scale. Similar to Figure 7, in this depiction, the slope *n* corresponds to the exponent of the relation of $f\left(\theta_{z}=\mathrm{const}.,\Delta \theta_{z}\right)\propto {\Delta \theta_{z}}^{n}$.

Two cases have to be distinguished. For initial angles $\theta_{z}$ close to $0{}^{{}^{\circ}}$, the relation can be approximated by an exponent of n=2:

(11) $$\lim_{\begin{array}{c} \theta_{z}\to 0{}^{{}^{\circ}}\\ \Delta \theta_{z}\to 0{}^{{}^{\circ}} \end{array}}f\left(\theta_{z},\Delta \theta_{z}\right)\propto \theta_{z}{\left(\Delta \theta_{z}\right)}^{2}$$

However, for $\theta_{z}>0{}^{{}^{\circ}}$, there is a bend in the subfunction. This leads to an exponent of n=1 with increasing $\theta_{z}$ and decreasing $\Delta \theta_{z}$. For $\theta_{z}$ close to $90{}^{{}^{\circ}}$, we can approximate:

(12) $$\lim_{\begin{array}{c} \theta_{z}\to 90{}^{{}^{\circ}}\\ \Delta \theta_{z}\to 0{}^{{}^{\circ}} \end{array}}f\left(\theta_{z},\Delta \theta_{z}\right)\propto \theta_{z}\left(\Delta \theta_{z}\right)$$

### 3.8. Scaling Behavior for Initial Angles Close to $0{}^{{}^{\circ}}$

First, we use the approximation of $f(\theta_{z},\Delta \theta_{z})$ for small initial angles $\theta_{z}$. Using Equations (9) and (11), we find:

(13) $$\mathrm{MRE}=\frac{2mB\theta_{z}{\left(\Delta \theta_{z}\right)}^{2}}{G^{\prime }\gamma^{2}V}$$

In the case with no magnetic field, the rotation angle $\Delta \theta_{z}$ can be assumed to be $\tan\left(\gamma \right)$. In the case with a magnetic field, the rotation angle will be smaller; however, a proportionality can still be assumed. With a small angle approximation, we can use:

(14) $$\Delta \theta_{z}\propto \tan\left(\gamma \right)\approx \gamma$$

Additionally, the magnetic moment *m* of the material over the volume *V* equals the magnetization $M_{p}\left(B\right)$ times the volume content $\phi_{p}$ of the used magnetic particles:

(15) $$\frac{m}{V}=M_{p}\;\left(B\right)\;\cdot \;\phi_{p}$$

The initial angle $\theta_{z}$ can be assumed to be proportional to the mean particle *z*-angle $\bar{\theta_{z}}$ of our tomographic data. $\bar{\theta_{z}}$ is a quantity with very large uncertainties; therefore, only tendencies can be reliably derived from the experimental data. In the case of the magnetic field, we ideally assume an inverse relationship of $\bar{\theta_{z}}\propto B^{-1}$.

(16) $$\theta_{z}\approx \bar{\theta_{z}}\propto \frac{1}{B}$$

The storage modulus of the matrix material has an impact on the initial angle $\theta_{z}$ as well as the rotation angle $\Delta \theta_{z}$. While the exact scaling behavior is not clear, the total scaling behavior can be expressed by an unknown exponent *n*:

(17) $$\theta_{z}{\left(\Delta \theta_{z}\right)}^{2}\propto {\left(G^{\prime }\right)}^{n}$$

Combining Equations (14) to (17) and (13), we find the following scaling behavior:

(18) $$\mathrm{MRE}\propto \frac{2M_{p}\;\left(B\right)\;\cdot \;\phi_{p}}{{G^{\prime }}^{\left(1-n\right)}}$$

### 3.9. Scaling Behavior for Initial Angles Close to $90{}^{{}^{\circ}}$

While most assumptions used for initial angles close $0{}^{{}^{\circ}}$ still apply for the scaling behavior for angles close to $90{}^{{}^{\circ}}$, because of the different scaling behavior with $\Delta \theta_{z}$, Equation (18) changes to:

(19) $$\mathrm{MRE}\propto \frac{2M_{p}\;\left(B\right)\;\cdot \;\phi_{p}}{\gamma {G^{\prime }}^{\left(1-n\right)}}$$

with *n* being the total scaling exponent of $\theta_{z}\left(\Delta \theta_{z}\right)\propto {\left(G^{\prime }\right)}^{n}$, analogous to Equation (17). In this case, there is a residue scaling behavior of the MRE with the shear strain γ.

### 3.10. Applicability of the Approximations

Since the initial angle $\theta_{z}$ is influenced by the magnetic flux density *B* and since the initial angle influences the scaling behavior of $f(\theta_{z},\Delta \theta_{z})$ with $\Delta \theta_{z}$, this leads to a complex interaction that influences the scaling behavior of the MRE. For soft materials and for high magnetic flux densities, Equation (18) applies, while Equation (19) applies for low magnetic flux densities and hard materials. In our previous study, we observed slight differences in the scaling behavior of the MRE with *B* between the two investigated degrees of swelling, which could be explained by this interaction.

Additionally, between these two degrees of swelling, different shear strains γ were used, with γ = 1% in the swollen and γ = 0.1% in the deswollen state. As our material has very different shear moduli in the two degrees of swelling, we aimed for the measurements to be within the respective linear viscoelastic plateau and for comparable shear stress τ. For the swollen state, higher shear strains were necessary for the torque to be above the detection limit of the rheometer, while for the deswollen state, the destruction of the samples caused by high shear strains had to be avoided.

If Equation (19) applies to the deswollen state, the change in shear strain γ impacts the determined scaling behavior of the MRE with $G^{\prime }$ of our empirical law. The scaling behavior was determined for B=500 mT, which was the highest magnetic flux density used. According to Table 3, in our tomographic data, the mean initial angles at B=380 mT were around $\theta_{z}=45{}^{{}^{\circ}}$ in the deswollen state. While lower angles can be expected at B=500 mT, the angles are unlikely to be close to $0{}^{{}^{\circ}}$.

On the other hand, since we used soft magnetic magnetite particles, the orientation of the magnetic dipole moment and the orientation of the long axis of the particles do not necessarily perfectly align and are likely to diverge to a certain degree to allow a better alignment of the magnetic dipole moment and the magnetic field. In this case, the orientation of the magnetic dipole moment is closer to $0{}^{{}^{\circ}}$, which would allow the use of Equation (18) even for harder materials. This needs to be experimentally verified by shear-strain-dependent rheological measurements. Since the exact scaling exponent *n* of $f\left(\theta_{z},\Delta \theta_{z}\right)\propto {\left(\Delta \theta_{z}\right)}^{n}$ is itself a function of *B*, these experiments also need to be performed at different magnetic flux densities.

If Equation (18) applies even in the deswollen state, in the case of n=0.5 as the total scaling exponent of $\theta_{z}{\left(\Delta \theta_{z}\right)}^{2}\propto {\left(G^{\prime }\right)}^{n}$ and for $M_{p}\;\left(B\right)\propto L\left(B\right)$, the scaling behavior of this theoretical approach equals the empirical law that we found previously with our rheological data:

(20) $$\frac{2M_{p}\;\left(B\right)\;\cdot \;\phi_{p}}{{G^{\prime }}^{0.5}}\hat{=}\frac{K_{p}\;\cdot \;L\left(B\right)\;\cdot \;\phi_{p}}{\sqrt{G^{\prime }}}$$

If Equation (19) applies, the scaling behavior of the MRE with $G^{\prime }$ of our rheological approach needs to be corrected. For either case, this energy-based approach can explain the observed scaling behavior with relatively simple assumptions.

### 3.11. Implications of the Proposed Mechanism

In our previous study, we discussed differences between the empirical law and scaling behaviors, which could be assumed a priori—for example, a scaling of the MRE with $L\left(B\right)\;\cdot \;B$ instead of a scaling solely with $L\left(B\right)$. However, this mechanism backed by tomographic data shows that the differences are a direct consequence of the initial angle of the particles and are based on only the rotation of the particles.

There is no interaction between the particles required, and no chain formation takes place. Differences between the scaling behavior of the MRE with *B* for different degrees of swelling can be explained by the complex interaction of *B*, $\theta_{z}$ and $\Delta \theta_{z}$. Since Equation (18) is only a proportional scaling law, a material constant $K_{p}$, which represents the particle–matrix-interaction, is still required to calculate the MRE of a given material system.

**Figure 9:**
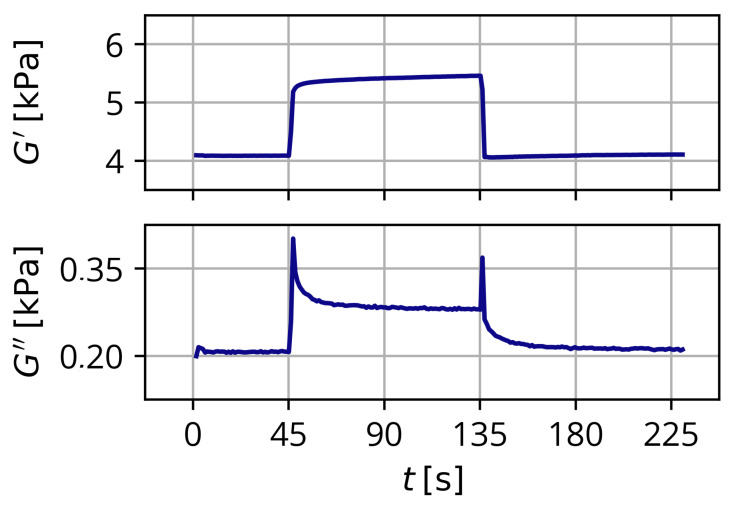
Exemplary measurement curves of the MRE for $G^{\prime }$ and $G^{\prime \prime }$ for a sample with $w_{s}$ = 30%, *c*_NaCl_ = 0 mol L^−1^ at $T=20\;{}^{{}^{\circ}}C$.

Exemplary measurement curves of the MRE are shown in Figure 9. In the case of the storage modulus, an almost instantaneous increase of the modulus with the activation of a magnetic field and an almost instantaneous decrease back to the base level with the deactivation of the magnetic field can be observed in the rheological experiments. Since the additional stored energy is the direct result of the magnetic force and is not a consequence of structural changes, the magnetorheological effect of the storage modulus shows almost the same dynamic behavior as the magnetic field.

In the case of the loss modulus, the observed MRE is the consequence of an increase of the dissipated energy when the sample is sheared. This can be linked to irreversible changes of the matrix due to the rotation of the particles. The rotation of the particles leads to a displacement of the matrix material in the vicinity of the particles and increased friction between the particles and the matrix material. Additionally, some local damage to the matrix must be expected. In our rheological experiments, we found a sharp instantaneous increase of the loss modulus with the activation of the magnetic field, which relaxed to an increased level compared to the base level.

After deactivation of the magnetic field, there was again a sharp increase followed by a relaxation of the loss modulus back to the base level. With our mechanism, the initial increase stems from the damage and displacement of the local matrix material surrounding the rotating particles. Afterward, there is a dynamic steady state with increased friction within the material due to the rotation of the particles. When the magnetic field is deactivated, displaced matrix material can return, which leads to the slow decrease of the loss modulus.

Within the precision of our measurements, we found no irreversible changes to the mechanical properties, which were significantly higher than the observed drift effects. Following this mechanism, only a reduction of the storage modulus could be expected, which would be the result of structural damage to the matrix material. Changes to the loss modulus could be either positive or negative, depending on how the structural damage impacts the dissipation of energy within the matrix during shearing.

The proposed mechanism is significantly different from what can be found in the literature. For example, Lopez-Lopez et al. proposed that induced anisotropy of the particles is strictly required for a positive MRE [10]. In their theoretical study, they assumed spherical magnetically soft particles, which led to no rotation of the particles. In our study, we used aspherical particles, which could explain the significant differences between the mechanisms. Additionally, nanocomposite hydrogels have a very specific structure and crosslinking mechanism, which could further account for the observed differences compared to other hydrogel systems [11].

Based on this mechanism, if a sample of our material is prepared within a magnetic field to align the particles during the synthesis of the gels, the resulting anisotropy should have no impact on the observed MRE as the particle interaction is assumed to be negligible for the MRE. As discussed, additional shear-strain-dependent measurements are necessary to check the scaling behavior of the MRE with $G^{\prime }$ and whether there is an impact of γ on the MRE. With such experiments, this mechanism could be further supported.

## 4. Conclusions

In this study, we investigated the scaling behavior of the magnetorheological effect of nanocomposite hydrogels with magnetite micro-particles through tomographic experiments and a very basic energy-based theoretical approach. We used salt as a deswelling stimulus, which considerably simplified the requirements of the sample chamber for the tomographic experiment. Rheological experiments indicated that, while the mechanical properties were not identical when salt was used as the stimulus instead of temperature, they were sufficiently similar to extrapolate between the hydrogels used in this study and our previous study.

Tomographic experiments were performed for varying particle contents and degrees of swelling for increasing magnetic flux densities. We found that particles inside our material exhibited only rotational and no translational movement. Additionally, the rotational movement was largely irreversible. Based on this knowledge, we developed a simple energy-based model for the magnetorheological effect of nanocomposite hydrogels with magnetite microparticles, which led to a scaling law that matches the previously found empirical law, requiring few approximations and assumptions.

Based on this mechanism, the specific scaling behavior is the direct result of the angle distribution of the magnetic particles and the impact of the magnetic flux density and the mechanical properties of the matrix material on the angle distribution. While mechanisms for the MRE in various systems typically involve movement, chain formation and interaction of the particles, our tomographic data as well as our mechanism show that this is not always required and that a MRE based solely on the rotation of the magnetic particles is possible. The proposed mechanism also explains the dynamic behavior of our magnetorheological measurements.

## Figures and Tables

**Table 1:** Composition of synthesized magnetorheological gels. $w_{s}$ and $\phi_{p,0}$ denote the mass and volume fraction of the particles in the initial state of synthesis.

$w_{s}$ [%]	$\phi_{p,0}$ [%]	$m_{\mathrm{pregel}}\;\left[g\right]$	$m_{\mathrm{initiator}}\;\left[g\right]$	$m_{\mathrm{particles}}\;\left[g\right]$
10	2.1	5	0.45	0.61
30	7.7	5	0.45	2.34

**Table 2:** The storage and loss moduli for gels, either deswollen by temperature (indexed *T*) or salt (indexed NaCl). The data for $G_{T}^{\prime }$ and $G_{T}^{\prime \prime }$ are from the batches from the previous study. The swollen state was measured at T= 20 °C and *c*_NaCl_ = 0 mol L^−1^ and the deswollen state at T= 40 °C and *c*_NaCl_ = 0 mol L^−1^. $G_{\mathrm{NaCl}}^{\prime }$ and $G_{\mathrm{NaCl}}^{\prime \prime }$ are from a new batch. The swollen state was measured at T= 20 °C and *c*_NaCl_ = 0 mol L^−1^ and the deswollen state at T= 20 °C and *c*_NaCl_ = 1 mol L^−1^.

State	$G_{T}^{\prime }\left[k\mathrm{Pa}\right]$	$G_{\mathrm{NaCl}}^{\prime }\left[k\mathrm{Pa}\right]$	$G_{T}^{\prime \prime }\left[k\mathrm{Pa}\right]$	$G_{\mathrm{NaCl}}^{\prime \prime }\left[k\mathrm{Pa}\right]$
Swollen	3.0±0.9	3.6±0.8	0.13±0.07	0.15±0.08
Deswollen	430±190	140±30	65±24	15±4

**Table 3:** Sample parameters, the mean particle *z*-angle ${\bar{\theta_{z}}}_{B}$ at B=380 mT and the ratio of the initial and the lasting change of the solid angle as calculated by Table 2.

$w_{s}$[%]	$c_{\mathrm{NaCl}}$[mol L${}^{-1}$]	$G^{\prime }$[kPa]	${\bar{\theta_{z}}}_{B}{[}^{{}^{\circ}}]$	$\frac{\Delta {\bar{h_{\Omega }}}_{0}}{\Delta {\bar{h_{\Omega }}}_{B}}$[%]
10	0	3.2±0.3	26.5	86
10	0	3.2±0.3	21.3	75
10	0.1	6.7±0.2	23.0	68
10	1	127±16	43.8	92
30	0	3.7±0.6	20.5	76
30	0.1	6.3±0.4	23.1	65
30	1	151±22	45.7	74

## Data Availability

The data presented in this study are available on request from the corresponding author.

## Abbreviations

The following abbreviations are used in this manuscript:

MRE	magnetorheological effect
NIPAAm	*N*-isopropylacrylamide
